# Pediatric deep burn management after split-thickness autologous skin transplantation: A comparative study

**DOI:** 10.1097/MD.0000000000027633

**Published:** 2021-11-05

**Authors:** Aba Lőrincz, Anna Gabriella Lamberti, Zsolt Juhász, András Garami, Gergő Józsa

**Affiliations:** a Department of Thermophysiology, Institute for Translational Medicine, Medical School, University of Pécs, 12 Szigeti Street, Pécs, Hungary; b Surgical Division, Department of Paediatrics, Medical School, University of Pécs, 7 József Attila Street, Pécs, Hungary.

**Keywords:** deep pediatric burn, hydrofiber dressing, silver foam, skin graft, Zinc-hyaluronic acid

## Abstract

Treatment of pediatric deep burns remains a challenge for healthcare personnel. After skin grafting, several treatment options are available, but comparative studies of the different options are scarce. Here, we compared the effectiveness of 2 postoperative dressings used to treat deep pediatric burns after split-thickness skin grafting.

At the Department of Paediatrics, University of Pécs, 16 children received skin transplantation after the deep second and third-degree injuries between January 1, 2012 and December 31, 2020 whose results have been analyzed, in this cohort study. We compared the traditionally used Grassolind or Mepitel net and Betadine solution (comparison group) with Aquacel Ag foam and Curiosa gel (intervention group).

Seven children were included in the comparison and 9 children in the intervention group. In the control group, the average number of anesthesia was 6.29, while the number of dressing changes was 4.29. After complete wound closure, the dressing's final removal was on the 13th day, while the mean length of hospitalization was 21.89 days. On average, in the intervention group, 3.56 anesthesia was induced, and 0.66 dressing changes were needed after transplantation. Complete healing (dressing removal) was on the 10th day, and the mean length of hospitalization was 12.38 days.

In the intervention group, the need for anesthesia significantly decreased by 43% (*P* = .004), and they required 84% fewer dressing changes after transplantation (*P* = .001). Moreover, the dressing could be removed 3 days earlier, and the length of hospitalization was reduced by 45% on average.

## 1 Introduction

Recent studies showed that the incidence of burn injuries in children is increasing in several countries, while pediatric burns still constitute a challenge for healthcare, even in the developed world.^[1]^ In severe forms, burns can also damage deeper tissues besides the skin. Children younger than 5 are at the highest risk because their reflexes are still developing. Furthermore, their skin is thinner than in adults, and they explore the environment without experience with hot subjects and surfaces.^[2,3]^ Without proper therapy, burn injuries can result in lifelong functional, aesthetical, and psychological complications, such as hypertrophic scarring, contractures, or post-traumatic anxiety disorders.^[4–6]^

In the treatment of thermal injuries, the first 24 to 48 hours are of crucial importance. Missed or inadequate interventions can increase the frequency, severity, and duration of the complications, resulting in an extended hospital stay and a higher cost of care.

Irreversible skin damage in burns is the primary cause of adverse consequences. Therefore, full recovery can only be expected if the integrity of the epidermis has been restored.

The severity and prognosis of a burn are determined by the depth, location, and area of the injury, along with the patient's age and general health.^[7,8]^ Deep partial burns (earlier known as II/2) impact the stratum reticulare in the dermis as well. The wound bed is numb with a blotched pale, white, or purple color. Without medical intervention, spontaneous recovery is eventual and often results in extensive hypertrophic scar development. Full-thickness (also known as third-degree) thermal injuries damage the entire skin, which becomes necrotized as well as painless, pale, and pearly. Spontaneous healing does not occur in this condition, and an operative approach is necessary to help the patients.^[7]^ In these severe forms of burns (ie, deep partial and full-thickness), the administration of prompt and effective treatment is of utmost importance.

Earlier clinical trials with the application of either Aquacel Ag foam or Curiosa gel found beneficial effects for each of these treatments individually in second-degree burns.^[9–19]^ However, according to the best of our knowledge, as of today, no data are available on the effect of combining these treatments in pediatric patients with deep second or third-degree burns after skin transplantation. Therefore, in the current study, we present the results obtained from skin grafted children with deep second and third-degree burn injuries treated simultaneously with Zinc-hyaluronic acid gel and a unique silver foam dressing.

## 2 Materials and methods

### 2.1 Design

We conducted a nonrandomized, single-center, comparative clinical trial at the Surgical Division, Department of Paediatrics, Medical School, University of Pécs, Pécs, Hungary. The intervention groups’ data were collected prospectively between January 1, 2015 and December 31, 2020. The children's characteristics were compared retrospectively with a control group, collected from patients with the same type of injuries who were treated at our clinic from January 1, 2012 to December 31, 2016.

The clinical application of the Aquacel Ag foam and Curiosa gel dressing combination was accepted and permitted by our medical board in 2010. The Hungarian Paediatric Surgery Committee approved the clinical study. Written informed consent was obtained from the patients’ guardians in the prospective arm. In the case of the retrospectively collected patient outcomes, permission could not be acquired in every instance; thus, the head of our medical team (GJ) took responsibility for the children's anonymization.

### 2.2 Participants

Between January 1, 2012 and December 31, 2020, 62 children (younger than 16-year-old) visited our clinic with deep partial or full-thickness burns treated with the combination therapies. Children with comorbidities (6 patients) or more extensive burns than 15% of the total body surface area (TBSA) (9 patients) and those who did not receive skin grafts (16 patients) were not included in the present study. We have excluded 15 patients from our registry due to missing photo documentation, thus, the injuries’ depth, grafted area, or wound closure time could not be verified. Attending the short-term (1-month) follow-up was also required for participating in this study, and the mid- and long-term control examinations are still ongoing. Finally, 16 children with deep burns were included in this study, 7 treated with the traditional and 9 with the modern methods. The available data limited the study size because no surgeon at our department could unreservedly apply the conventional dressings after observing the advantages of the contemporary approaches.

### 2.3 Comparative treatment group

**Grassolind gauzes** (Hartmann, Germany) are widely used as inexpensive, paraffin impregnated dressings made of open-weave cotton cloth. These dressings are non-medicated and can be safely applied without any sensitizing effects after prolonged use. Their primary function is to create a temporary barrier between the host and the environment, thereby also preventing fluid loss.

**Mepitel** (Mölnlycke Health Care, Sweden) is a two-sided dressing with a silicone wound contact layer. It was designed to be quick and less painfully removable without causing damage to the regenerating skin. It also seals the wound margins to protect the skin from damaging leaks and maceration, while its perforations allow the exudate to pass through into a secondary absorbent dressing.^[20,21]^

**Betadine** (Egis Gyógyszergyár Zrt., Hungary) is an antiseptic solution. Its active ingredient is povidone-iodine, which has a broad-spectrum antimicrobial effect.

These traditionally used dressings are suitable for covering skin defects while treating burn injuries and managing the skin grafts in the post-transplantation phase. However, none of them has any antibacterial properties, which is why they were combined with Betadine in our institution.

### 2.4 Intervention treatment group

**Aquacel Ag** (ConvaTec, USA) foam is a Hydrofiber dressing consisting of a superficial polyurethane waterproof layer and a multi-layered absorbent surface containing 1.2% ionized silver. The dressing absorbs the wound secretion as the Hydrofiber layer transforms into a gel, facilitating wound-humidification and closure while protecting against infections.^[10,11,13,14,16,18,19]^

We have combined this silver-foam dressing with **Curiosa gel** (Richter Gedeon Nyrt., Hungary). Its main component is Zinc-hyaluronic acid that promotes cell regeneration; therefore, it contributes to faster wound closure. Moreover, zinc has antibacterial effects, while the gel formulation helps prevent the adhesion of the silver foam to the base of the burn wound.^[9–11]^

### 2.5 Treatment protocol

**Figure 1:**
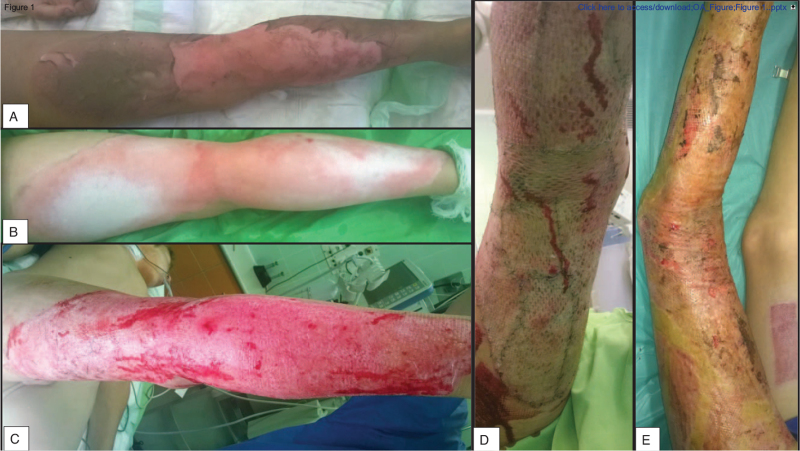
Timeline of an intervention treated burn patient's management. It shows the state of the burn A) upon admission, B) after cleaning the injury, C) following necrectomy, D) subsequent to STSG, and E) at the final dressing removal. STSG = split-thickness skin graft.

After the burn injury, each child in the study received primary care before hospital admission. Most patients were transported to our department after their wounds were cooled with running tapwater during first aid, received temporary coverage, and pain medications in the ambulance. All children were assessed by an experienced burn specialist in our department, who also determined further therapeutical steps (Fig. 1A).

**Figure 2:**
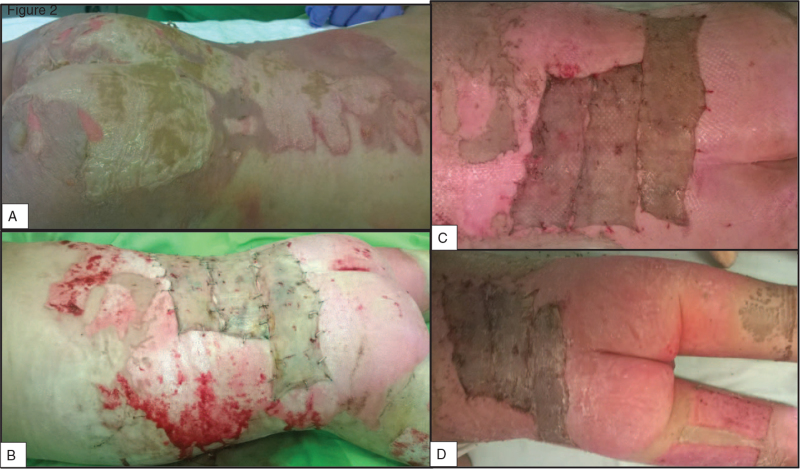
Photo documentation of a control group patient's recovery. A) Admission picture, B) after STSG, C) the state of the injury at a dressing change, and D) discharge photo. STSG = split-thickness skin graft.

After disinfecting and cleaning the burn site (Figs. 1B and 2A), the children required bullectomy and tangential necrectomy (Fig. 1C), which was performed in general anesthesia induced with Calypsol (ketamine – Richter Gedeon Nyrt., Hungary); afterward, we determined their need for transplantation. If we could safely ascertain the depth of the injury after the debridement, we simultaneously performed split-thickness skin graft (STSG) transplantation (Figs. 1D and 2/B). We re-examined the wound's base 2 days later in case of uncertainty, and then we carried out the operation if required.^[22]^ After the transplantation, we applied Grassolind, or Mepitel nets, combined with Betadine solution in the comparison group, whereas Aquacel Ag with Curiosa in the treatment group.

**Figure 3:**
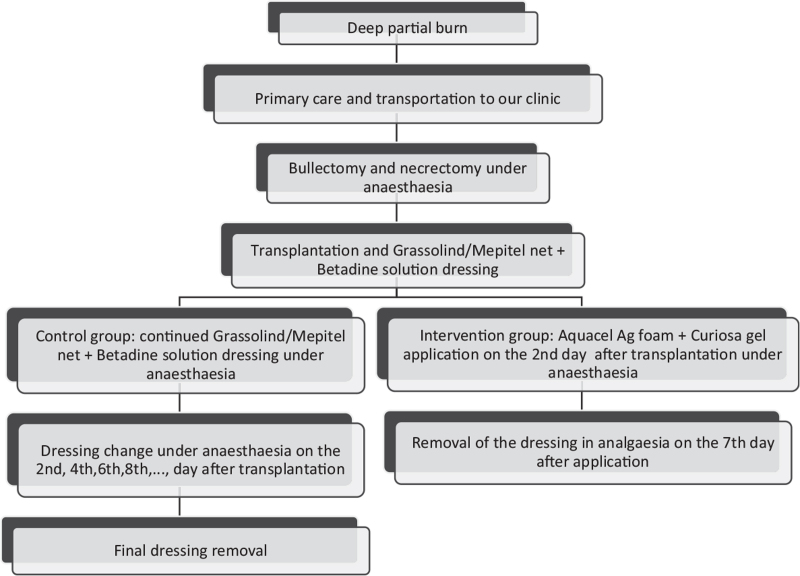
The treatment protocol for the 2 groups in this trial.

In the comparison group, the application, changing (every other day after the transplantation (Fig. 2C)), and final removal of the dressings (Figs. 2D and 3) were all made in narcosis. General anesthesia was required due to the common complication of dressing adherence to the wound bed. In these cases, the dressing's removal resulted in the tearing up of the still regenerating epithelium, bleeding, and skin loss. A previous study confirmed that a moist or wet environment is beneficial for the burned skin's healing process.^[23]^ Consequently, these dressings’ frequent changes were necessary because povidone-iodine-soaked gauzes were usually dehydrated after 2 days in our clinical experience and lost their antibacterial efficacy.

In earlier studies about pediatric superficial second-degree burns, we have found that the silver foam dressing can be easily removed after complete reepithelialization – it is like the scab's spontaneous separation from the wound bed – therefore, it is painless.^[10,15]^ As a result, we applied only the first dressing under general anesthesia in the intervention group. Narcosis was not necessary afterward, except when the dressing was contaminated or excessive fluid discharge from the wound was observed. Seven days after the initial application, we removed the foam dressings in general analgesia with diclofenac (Cataflam; Novartis Hungaria Kft., Hungary) or under the effects of the anxiolytic midazolam (Dormicum; Egis Gyógyszergyár Zrt., Hungary) or both medications (Figs. 1E and 3).^[8,24]^

In both groups, the graft donor sites were treated with the control treatment dressings, but only the evaluation of the transplanted areas was done in this study.

**Figure 4:**
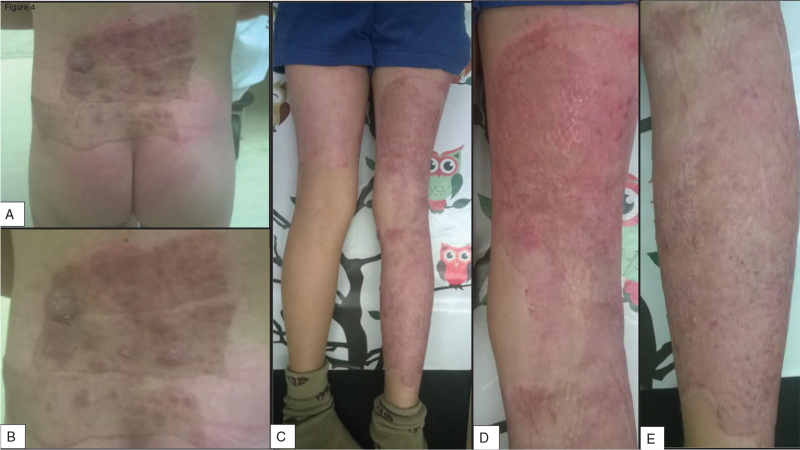
One-month control appointment results of a patient from the (A–B) comparison and (C–E) intervention group.

A month later, the patients were recalled for a control appointment where their injury was re-examined for possible complications,^[4–6]^ but fortunately, none of the children suffered any (Fig. 4).

### 2.6 Outcomes and demographics measured

A photograph was taken of every patients’ burn before applying the first dressing and after that, at every dressing change until complete wound closure. The children were evaluated based on 9 aspects. We analyzed the patients’ demographic data, such as sex and age distribution, etiology, grafted surface area, and the severity of the burns. Our primary outcomes were the average days required until complete healing, the number of required anesthesia and dressing changes after skin transplantation, as well as the total days of hospitalization.

### 2.7 Statistical analysis

Statistical analyses were performed with Statistics Kingdom calculators available at https://www.statskingdom.com. Welch *t* test was used for continuous variables because of the unequal group sizes and variances, while the Mann–Whitney *U* test was used if the data had discrete variables. With a confidence interval of 95%, probabilities of less than .05 were considered significant. Consultations with a biostatistician were held to confirm our choice of tests.

## 3 Results

### 3.1 Distribution by sex

**Figure 5:**
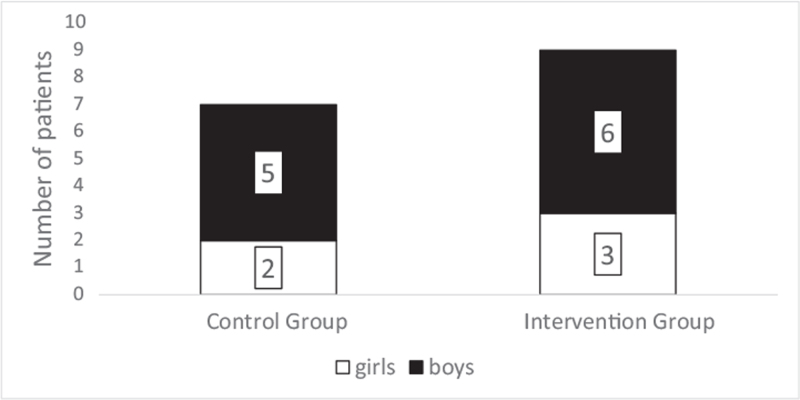
The sex distribution of patients.

Five boys and 2 girls were included in the comparison group, while 6 boys and 3 girls were in the intervention group (Figure 5). Thus, the ratio of boys was 71.43% in the control, and 66.67% in the intervention, while it was 68.75% in the 2 groups combined.

### 3.2 Distribution by age

**Figure 6:**
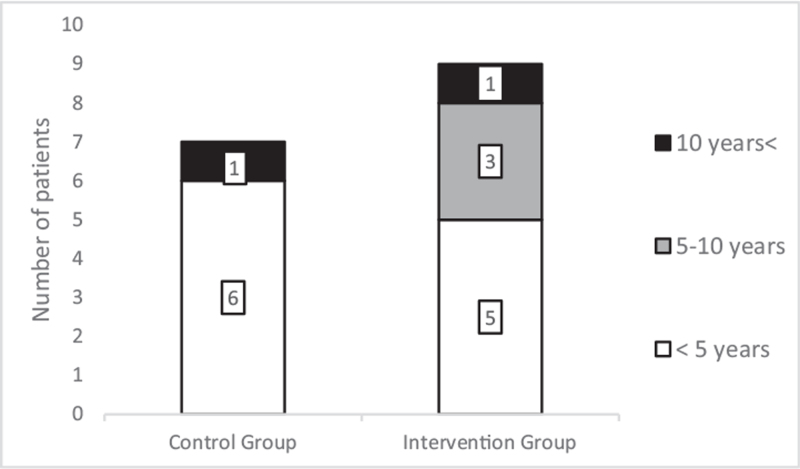
The patient's distribution via their age.

The patients’ mean age was 3.00 years (standard deviation, SD: 2.56; range: 1–12 years) at the time of the accident in the control group, while it was 4.88 years (SD: 4.38; range: 1–16 years) in the intervention group. The distribution of the patients’ age in the groups; younger than 5, between 5 and 10, and older than 10 years old is shown in Figure 6. Only 1 child was older than 10years in both groups. Children younger than 5 years old had the highest incidence rate for deep burns (68.75% of all cases).

### 3.3 Distribution by etiology

**Figure 7:**
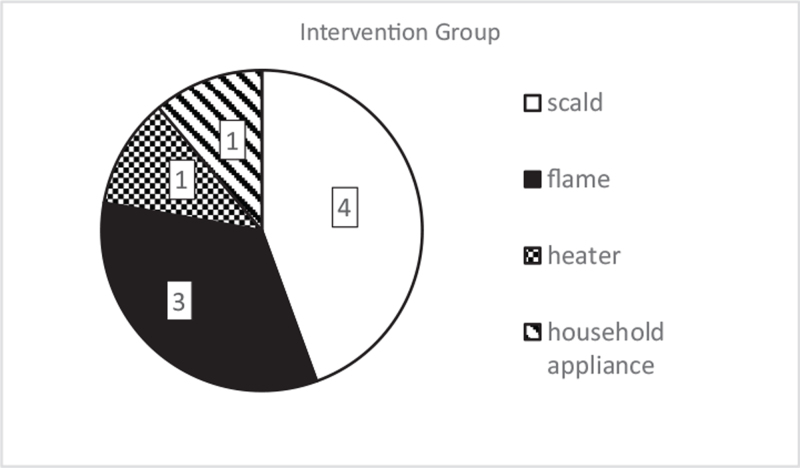
The number of burn injuries based on etiology in the treatment groups.

We also studied the cause of burn injuries. Every case was due to an unintentional accident; the possibility of an intentional insult did not arise regarding any included children. In the comparison group, all thermal injuries occurred due to scalding (Fig. 7). The intervention group included 4 children with scalds (44.44%), 3 with flame (33.33%), and 2 with contact burns (22.22%). Of the latter, 1 was caused by a heater and the other by a household appliance. The most frequent etiology was scalding injury occurring in 68.75% of all patients and 89.89% in younger than 5-year-old children.

### 3.4 Mean grafted TBSA%

**Figure 8:**
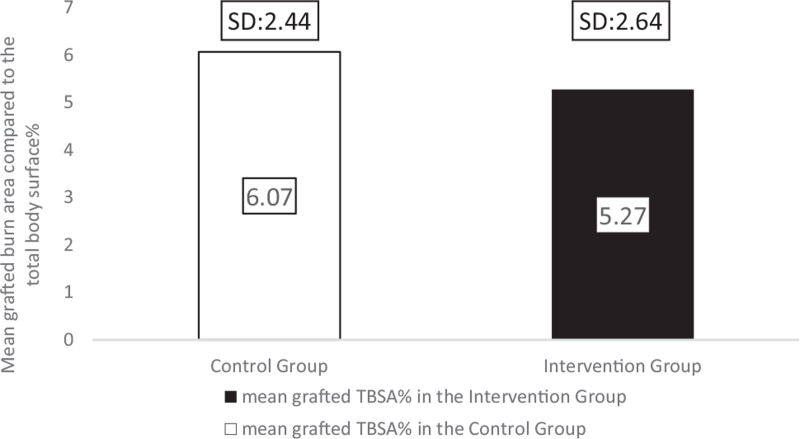
The mean grafted burn areas in the analyzed groups.

The extent of the burn injuries that received STSG was measured using the Lund–Browder schema,^[7,28]^ based on which we have calculated the percentages of the burned area compared to the TBSA. In the control group, the grafted burn injury's extent was 6.07 TBSA% (SD: 2.44; range: 2–8) and in the intervention group, the grafted TBSA% was 5.27 (SD: 2.64; range: 2–8) (Fig. 8). Although the mean TBSA% of the intervention group is 13.18% lower than that of the comparison group, the difference was not statistically significant (*P* = .57).

### 3.5 The severity of the burns

We used the American Burn Association classification to determine the burns’ severity.^[29]^ Five out of 7 burns were major (71.43%) in the comparison group: 4 suffered more than 10 TBSA%, mostly superficial partial-thickness burns, and 1 had a facial burn injury. In the intervention group, 7 out of the 9 children had major injuries (77.78%). Three had second-degree burns of more than 10 TBSA%, 2 had hand and facial burn injuries, and 2 suffered burns of the feet, with 2 patients having a combined reason for increased severity. The rest of the second-degree burns were moderate in both treatment groups because the affected area was 5 to 10 TBSA%.

We have also compared the ratio between grafted third and deep second-degree burns, and in the comparison group, there was only 1 child (14%) with full-thickness injuries. At the same time, there were 3 children (33%) in the intervention group.

### 3.6 The mean time to reepithelialization

**Figure 9:**
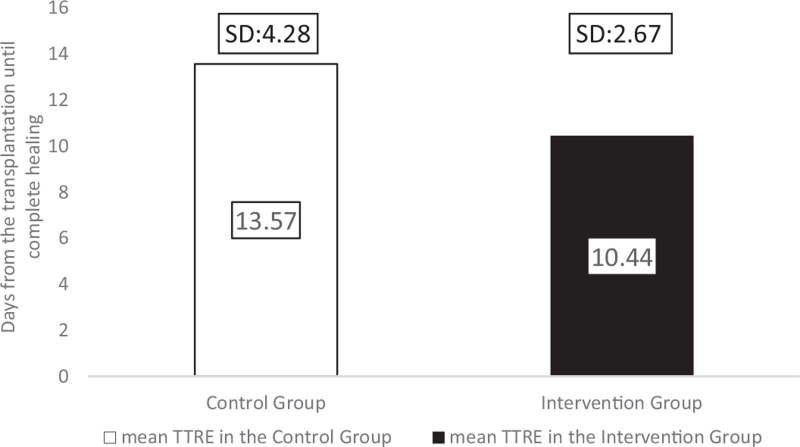
The average TTRE in the 2 groups. TTRE = time to ReEpithelization.

We defined time to reepithelialization (TTRE) as the average duration from transplantation of the skin graft until the transplanted skin's complete wound closure without wound leakage. Patients in the comparison group needed an average of 13.57 (SD: 4.28; range: 8–18) days until reepithelialization, whereas in the children in the intervention group, TTRE was only 10.44 (SD: 2.27; range: 7–15) days (Fig. 9). Although the difference was not statistically different between the 2 groups (*P* = .246), the healing time was 23% shorter after Hydrofiber silver-foam with Zinc-hyaluronic acid gel dressings than in the comparison group. To adjust for the slight difference in the burned surface area, the healing time by area (TTRE/TBSA%) was calculated to measure how long it takes for a hypothetical 1 TBSA% burn to regenerate. The comparison group required 2.24 days, while the intervention group needed 1.98 days for a 1 TBSA% wound closure, which is an 11.38% time reduction with the intervention treatment.

### 3.7 The average number of anesthesia required

In the comparison group, the dressing was changed every other day, which required general anesthesia in the operating theatre with an average of 6.29 times (SD: 1.28; range: 4–8).

**Figure 10:**
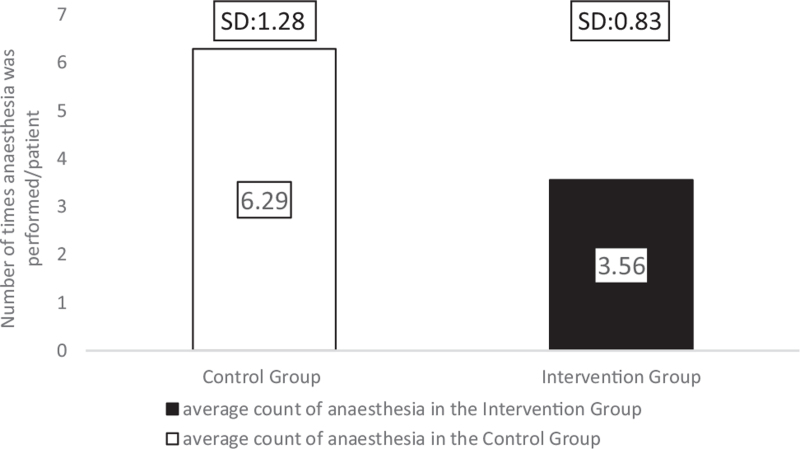
The mean number of times anesthesia in the 2 treatment groups.

The Hydrofiber dressings combined with Zinc-hyaluronic acid gel dressing were applied on the second day after the skin graft transplantation under general anesthesia. Afterward, further dressing changes were not needed, except in case of contamination or excessive wound leakage. Seven days later, the dressing was removed in general analgesia or under the effects of anxiolytic or both medications. In the intervention group, anesthesia was needed an average of 3.56 times (SD: 0.83; range: 2–5), which was significantly less than what was required for the comparison group patients (*P* = .004) (Fig. 10).

### 3.8 The average number of dressing changes after transplantation

**Figure 11:**
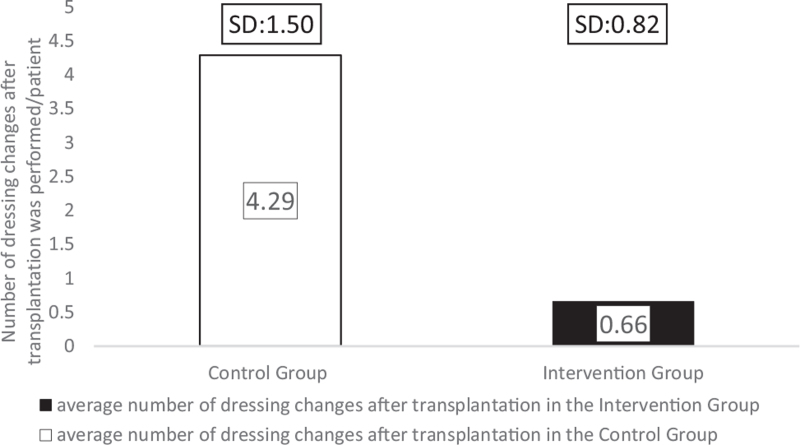
The mean number of dressing changes after transplantation performed in the groups.

A significant difference was found in the average number of dressing changes after transplantation between the treatment groups (*P* = .001). The intervention group only got their dressing combinations first applied 2 days after the transplantation, when the grafts’ take was confirmed. Therefore, the reapplication rate was measured in both groups after that moment in the patients’ management. There were, on average, 4.29 dressing changes (SD: 1.50, range: 2–6) in the comparison, whereas only 0.66 changes were required (SD: 0.82, range: 0–2) in the intervention group (Fig. 11). Thus, the frequency of dressing changes was reduced by 84.34%, resulting in less discomfort for the children. Consequently, the need for healthcare professionals and operating theatres were also reduced.

### 3.9 Mean length of hospital stay

**Figure 12:**
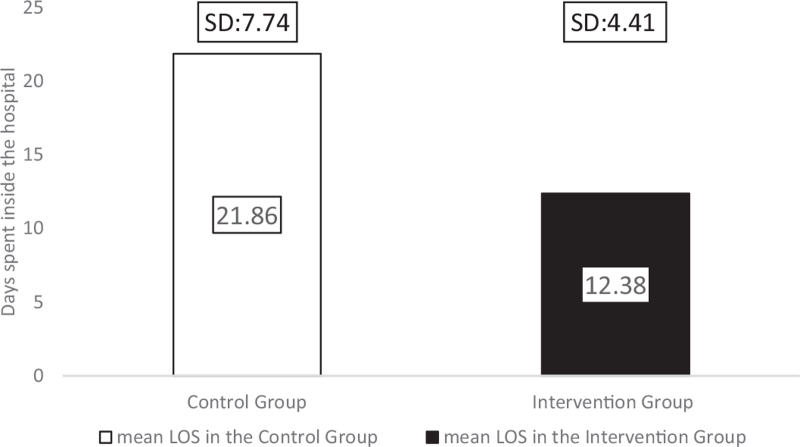
Length of stay inside the hospital, measured in days.

The duration of time spent inside the hospital is an essential factor for the child, family, and healthcare personnel. Our study compared the 2 treatment options and found a tendency for reduced length of hospital stay (LOS) in the intervention group (*P* = .055). Children in the comparison group spent on average 77% more time in the hospital than intervention group patients. The mean LOS for the comparison group was 21.86 days (SD: 7.74; range: 12–35), while children in the intervention group spent 12.38 days (SD: 4.41; range: 5–19) in hospital (Fig. 12). From this analysis, we had to exclude 1 patient from the intervention group. Even though the child's grafted wounds healed after 13 days, the patient spent 46 days in the hospital due to reasons independent from the burn injury. For clarity, without excluding the patient, the mean LOS in the intervention group would have been 16 days (SD: 10), which is still markedly lower than the LOS in the comparison group.

It is critical to highlight that in the intervention group, we were able to discharge multiple patients before the final dressing removal – after we have instructed the children and their parents on how to protect the dressing.

The summary of our findings is shown in Table 1.

**Table 1:** The summary of results.

	Control group			Intervention group	
No. of patients	7			9	
Outcomes	Mean	SD (range)	*P* value	Mean	SD (range)
Age (years)	3	3.7 (1–12)	.398	4.89	4.38 (1–16)
Grafted TBSA(%)	6.07	2.44 (2–8)	.57	5.27	2.64 (2–8)
TTRE (days)	13.57	4.24 (8–19)	.246	10.44	2.27 (7–15)
TTRE/TBSA% (days)	2.24		1.98		
DC (n)	4.29	1.5 (2–6)	.001	0.67	0.87 (0–2)
No. of anesthesia (n)	6.29	1.28 (4–8)	.004	3.56	0.83 (2–5)
LOS (days)^∗^	21.86	7.74 (12–35)	.056	12.38	4.41 (5–19)

TBSA% = total body surface area, TTRE = time to ReEpithelization, DC = dressing changes after transplantation, LOS = length of stay.

∗ The intervention group only contains 8 children in the LOS analysis.

## 4 Discussion

Predominantly boys suffered burn injuries in our study (68.75%). The grafted burn area was similar in all children (range: 2–8 TBSA%), with an average of 6.07 TBSA% in the comparison and 5.27 TBSA% in the intervention group.

Large population studies found correlations between age and the burn location, and they have discovered age-specific injury mechanisms.^[25–27]^ Under the age of 5, the typical causes included pulling hot liquid placed at a height onto themselves and directly touching the heater, while in children older than 10 years, flame-related wounds were dominant. Since young children's withdrawal reflexes are not yet fully developed, a moment of “freezing” is often present after coming in contact with heated surfaces, which increases the duration and severity of the burn.^[2,25–27]^

In our study, primarily children younger than 5-year-old suffered from deep burns (68.75% of all cases), of which 89.89% had scalding-related accidents. These results are in accordance with the results provided by international trials.^[1,25–27]^

The Hydrofiber dressing's unique layered design could interact with the wound exudate, and it provided a micro-environment that was optimal for healing for at least a week.^[30,31]^ The silver ions, in addition to zinc and povidone-iodine, facilitated the dressing's antimicrobial effectivity^[9,30]^ because none of the burn sites became infected. Consequently, we have found a significant reduction in the average number of dressing changes after transplantation in the intervention group (*P* = .001). In the comparison group, patients required 4.29 dressings, whereas only 0.66 changes were needed for children with the intervention treatment. The average number of anesthesia was also significantly reduced in the group treated with Hydrofiber dressings combined with Zinc-hyaluronic acid gel (*P* = .004) due to the interventions’ lower number of dressing changes and less traumatizing separation from the wound bed. Patients in the Grassolind or Mepitel net and Betadine solution group needed narcosis 6.29 times on average, whereas in the intervention group, it was only required 3.56 times. The TTRE was 13.57 days in the comparison group, while it took only 10.44 days in the intervention group. The faster healing times and fewer dressing changes explain the difference in the LOS; on average, patients spent 21.86 days inside the hospital in the comparison group, whereas only 12.38 days in the intervention group.

Limitations of our study must also be mentioned. We could not collect sufficient data from the patients’ pain levels and about the healthcare costs, retrospectively. However, we are confident that the less frequent dressing changes and anesthesia in the intervention group also caused less stress and discomfort to the children. These benefits for the patients, at the same time, can also reduce the need for hospital staff and operating rooms, thereby reducing the costs of healthcare. It must be emphasized that the patient population in our trial was small, and we collected patients’ data from a single center. Future trials are needed to confirm our results and ultimately to better treat and improve children's life quality with severe burn injuries.

In conclusion, our results showed that utilizing modern Hydrofiber dressings combined with Zinc-hyaluronic acid gel on children's burn wounds required 84% fewer dressing changes after transplantation compared to the traditionally used dressings at our clinic. Patients treated with the intervention did not require additional narcosis for the dressing changes (after the initial application); therefore, we could reduce the need for anesthesia by 43%. The children in the intervention group also had 23% faster-wound closure; thus, 45% shortened hospital stay, which data further support the benefits of the applied treatment in the intervention group.

## Acknowledgments

The authors would like to appreciate and thank all the healthcare providers at the Paediatric Department of Pécs for their rigorous and conscientious efforts, especially Flóra Varga, MD, who participated in data curation and investigation. We are genuinely grateful to the Department of Biostatistics at the Institute for Translational Medicine, Pécs, Hungary, for their statistical methodology guidance. We want to express gratitude to Péter Hegyi, MD, PhD, DSc for funding acquisition.

## Author contributions

AL participated in the data curation, formal analysis, interpretation, and visualization of the data as well as writing the original draft and reviewing and editing the article. GJ was involved in the conceptualization, methodology, investigation, project administration, and supervision processes in addition to reviewing and editing the article. AG took part in project administration, supervision, and reviewed and edited the article. AGL was involved in the investigation, formal analysis, and validation. ZJ reviewed and edited the article was involved in the investigation. All authors read and accepted the final manuscript.

**Conceptualization:** Gergő Józsa.

**Data curation:** Aba Lőrincz.

**Formal analysis:** Aba Lőrincz, Anna Gabriella Lamberti.

**Investigation:** Anna Gabriella Lamberti, Zsolt Juhász, Gergő Józsa.

**Methodology:** Gergő Józsa.

**Project administration:** András Garami, Gergő Józsa.

**Supervision:** András Garami, Gergő Józsa.

**Validation:** Aba Lőrincz, Anna Gabriella Lamberti, Zsolt Juhász, András Garami, Gergő Józsa.

**Visualization:** Aba Lőrincz.

**Writing – original draft:** Aba Lőrincz.

**Writing – review & editing:** Aba Lőrincz, Zsolt Juhász, András Garami, Gergő Józsa.
